# Items, Selections and Remarks

**Published:** 1869-11

**Authors:** W. W. Miner


### Items, Selections and Remarks.

BY W. W. MINER, A. B.

Among others, the following physiological points, descriptive of a person who was supposed to have been murdered, were determined by Drs. Mattock and Murphy, of St. Paul, Minn., from an examination of the remains exhumed after two years interment. The remains were those of a male, whose death was occasioned by violent means, which were two charges of shot. The deceased had a bifid spine, was five feet two inches in height, was left-handed, was knock-kneed, was between the ages of eighteen and twenty-five, was not a hard working man, wore a light mustache, short chin whiskers and a small tuft of side whiskers, and that the deformity of the spine was congenital. An interesting and more extended account of the matter is given in the October number of the *Psychological Journal.*——Prof. Mantegazzi, an Italian physiologist, lately spent sixteen hours in continued watching at the microscope for the appearance of organic life in an infusion he had prepared for the purpose. We think that these theorists who watch, will as well wait, also, for this link of the chain.

The *Humboldt Medical Archives*, of St Louis, acting on the principle that "science is cosmopolitan and should know no locality or nationality;" and on the event of the consolidation with it of the *Reporter*, has adopted the simple title, "The Medical Archives."——An American Journal of Syphilography and Dermatology is to appear, edited by Dr. M. M. Henry.——It was stated, by mistake, in the *Medical Gazette*, of Vienna, that Prof. Billroth, in an operation for ovariotomy, left a sponge behind in the abdomen, and that the patient died of peritonitis. It appeared that Prof. Braun was the operator, and that the operation was successful. Prof. Billroth has sued the journal for slander, and recovered damages to the amount of one hundred florins. The great opposition Prof. Billroth, a Prussian, has met with among the Austrians at Vienna, is given as an excuse for the rather unprofessional procedure.——James Campbell, of Boston, is publishing a reprint of *The Practitioner*, an English journal, edited by Drs. Francis E. Anstie and Henry Lawson.

The Decimal system of weight and measure has been adopted in the Austrian Pharmacopeia.——The British Association for the Advancement of Science has given £30 to Dr. Richardson for an investigation of the physiological action of organic compounds; and, to Dr. Gamgee, £15 for researches on the heat developed in the arterialization of the blood.——Gov. Claflin, of Massachusetts, has appointed a State Board of Health, of which Dr. Henry Bowditch has been elected President, and Geo. Derby, M. D., Secretary.——The New York Association for the Advancement of Science and Art has addressed a communication to the Board of Education, urging the importance of the physical and hygienic education of the pupils of our public schools.——Illinois is about to erect two Insane Asylums, one at Elgin, the other at Springfield. On November 10th, there was to be a meeting of the three boards of trustees of lunatic asylums of the State, and a number of invited experts, to discuss the relative value of the family, and congregate systems in the treatment of insane.

G. Von Liebig, having experimented on the physiological effects of compressed air, observes that: "The number of inspirations in an atmosphere submitted to a high pressure, when the animal has become accustomed to it, does not differ much from that which occurs ordinarily. The quantity of air breathed, and of carbonic acid exhaled, is identical with the normal quantities of the same.——Prof. W. Gibbs, M. D., of Harvard Coll., describes a new class of compounds with an acid of sulphur yellow color, derived from uric acid, which he has named stryphnic acid from its astringent bitter taste.

Part of the German Hospital which is being erected on 77th St., New York, has just been opened for use. The building, besides a basement and cellar, is three stories in height. Steam is used for heating, the sash of the windows is double-glazed, and the arrangements for ventilation are very complete. The cost of the building is $190,000. The medical board of the hospital consists of Drs Krackowizer, Lolman and Cinswer. The resident and visiting physicians number more than twenty, and the patients are of every nationality.——The Manhattan Eye and Ear Hospital, which was incorporated by the last legislature, have leased the building No. 233 East 34th St., and will open the institution this month. The corporation owns a lot, where it is intended eventually to build. Drs. Roosa, Agnew and Loring have been elected surgeons to the hospital.

Ligature of the aorta was recently performed in Edinburgh by Dr. Watson, on account of secondary hemorrhage from the common iliac artery after its ligature. The patient lived sixty-five hours after the operation. Ten cases only of this operation are recorded, in one of which the patient lived ten days subsequent to the operation.——Dr. Stanley, of Texas, says that he has used hypodermic injections of quinine hundreds of times, employs a solution containing thirty-two grains to the ounce of aq. distil., and finds that the alkaloid quinia is preferable to the sulphate, and that two grains of it, hypodermically, is equivalent to six grains by the mouth.

Prof. Von. Græfe has operated on W. H. Milburn, the blind lecturer, so that he can now appreciate light but cannot distinguish form, and next year the operation is to be repeated—with, it is hoped, complete success. Meanwhile the patient is lecturing in this country on "What a Blind man saw in pursuit of Sight."——Sir Henry Holland, Bart., a distinguished English physician, son-in-law of Rev. Sydney Smith, and formerly physician to Queen Caroline, is making a tour through the west and north-west part of our country. accompanied by his son, and by Hon. Wm. Evarts. Dr. Holland's narrative of former travels in Albania, Thessaly and Greece, are referred to in Lord Byron's works. He received, on arriving here, a cable telegram, giving the sad intelligence of the death of his son, aged nine years, by drowning the day after he embarked. This is said to be the eighth of Dr. Holland's visits to this country, with which he is much pleased. ——Peter Mark Roget, M. D., F. R. S., a distinguished physician and author, died at London in the ninetieth year of his age, Sept. 17th. He is best known to the American public as the author of "Roget's Thesaurus of English Words and Phrases," which was first published in Boston, in 1854. He wrote a number of scholarly treatises on scientific subjects, and was of marked eminence in his standing as a physician.

Dr. Chas. A. Shæffer has returned home, after spending two years and a half in scientific study in Germany, and has been appointed Professor of Analytical Chemistry in Cornell University.——Dr. C. D. Palmer, of Louisville, has been appointed to the professorship of Obstetrics and Diseases of Women and Children in the University of Louisville, the chair recently occupied by Theophilus Parvin, M. D.

Claude Bernard, the celebrated physiologist, has been called, by Napoleon III., to the French Senate. He is now, besides Academician and Senator, Professor of General Physiology at the Museum, Professor of Experimental Medicine at the College de France, Annual President of the Academie des Sciences (l'Institut,) Life President of the Societe de Biologie, Member of the Academy of Medicine, Commander of the Legion of Honor. Twenty-five years ago, Claude Bernard was an apothecary's assistant in a country town.—*Medical Record.*——J. Campbell Shorb, M. D., Prof. of Physiology in the Toland Med. Coll., San Francisco, Cal., in his introductory address to the students on "Benevolence in Medicine," concludes with these words: "The student of medicine who, in the consideration of quinine, opium, and chloroform, cannot discover abundant reason for gratitude to the good God above us, nor reason for devotion to medicine, had better leave these halls; or staying, pray that the obscurity of his soul may depart, and that his heart be aroused to a sense of the majesty and beneficence of the noblest of all human sciences."—*Cal. Med. Gazette.*——Dr. Bruce Jones, of London, Secretary of the Royal Institute of Great Britain, is writing a biography of Michael Faraday. ——Nebraska has organized a State Medical Society.——The next International Medical Congress will be held at Vienna.——D. Appleton & Co. announce a translation of "*Vogel on Diseases of Children,*" by H. Raphæl, M. D., a work which has been published in six different languages, and has reached four English editions.

Dr. Hay, of Philadelphia, publishes in the *Reporter* of Nov. 6th, an important and interesting account of the use for injections of "The Long tube in Intestinal Obstructions."——W. Scott Thorne, M. D., of San Jose, reports, in the *Cal. Med. Gazette*, a case of fatal hemorrhage produced during the operation of filling glass bottles with carbonic acid water, in which the pressure the bottle is made to sustain, is 125 lbs. per inch. A piece of a fractured bottle severed the external and internal jugular veins, the superior and inferior thyroid, and vertebral arteries, and also penetrated the left pleura.——Geo. M. Butler, M. D., of New York, publishes in the *N. Y. Medical Journal* a report of a case of chronic pneumonia treated successfully with oxygen inhalations.——M. Chassaignac has performed amputation of the thigh twice by means of the ecraseur and saw, but does not recommend this manner of operation generally.——Dr. Finnell presented, at a meeting of the New York Pathological Society, Sept. 22d, a specimen of a fatty heart, having a rupture over one-half an inch in length, in a portion of the left ventricle one-half an inch in thickness. The patient was run away with, and was found in the wagon in a dying condition, without any apparent external injury, and it was decided that the lesion of the heart was caused by fright.

A new organic principle has been extracted from the sugar-beet, which yields a beautifully crystallized salt with acids, and is named "*betain.*"——M. Guyot has determined, from an extended series of experiments, that coralline dye is not a poison, either when taken into the stomach or absorbed into the blood.——It is said that one-half the caffeine of coffee disappears in the process of roasting.——Coiguet, of Paris, says that, for purposes of anatomy, sulphurous acid gas removes the earthy material of bone more effectually than hydrochloric acid does.——Fruit stains may be removed from clothing by wetting the spot with a solution of hyposulphite of soda, and then sprinkling on tartaric acid ——Prof. Schutzenbuger is reported to have discovered a new sulphur acid formed by the action of zinc on sulphurous acid, which he names "hydrosulphurous acid," and symbolizes as SO, HO. It has a reducing power equal to that of nascent hydrogen, bleaches indigo, and forms a characteristic salt with soda.

Dr. Wm. M. Wood, of Maryland, who has been in the Naval service forty years, and who is thoroughly alive to the matters at stake in this department, has been appointed Chief of the Bureau of Medicine and Surgery of the U. S. Navy.——Profs. Hebra and Sigmund have, for twenty years, been professors extraordinary at Vienna without recompense, and at length, says the *Union Medicale*, they are named professors in ordinary.——Nelaton has performed ovariotomy sixteen times' nine of which cases were successful. In the case of unilocular cysts, with contents of a quite fluid character, he uses an injection of iodine, which he thinks acts in the same manner as it does in the cure of hydrocele.——Dr. G. M. Beard, of New York, in a letter to the *Record*, says: "Next to Humboldt, the scientific name which is dearest to the masses of the people in Berlin, is that of Græfe."

